# Supplementary material for: Fano interference of photon pairs from a metasurface
Source: Light Sci Appl. 2025 Oct 17;14:371. doi: 10.1038/s41377-025-01998-5 (PMC12534599; doi:10.1038/s41377-025-01998-5)
Supplement: Supplementary file 1 — Supplementary information for: Fano interference of photon pairs from a metasurface [file 41377_2025_1998_MOESM1_ESM.pdf]

Supplementary information for: Fano interference of photon pairs from a metasurface

Jiho Noh,[1,2] Tomás Santiago-Cruz,[1,2] Chloe F. Doiron,[1,2] Hyunseung Jung,[1,2] Jaeyeon Yu,[1,2] Sadhvikas J. Addamane,[1,2] Maria V. Chekhova,[3,4] and Igal Brener*[1,2]

[1]*Sandia National Laboratories, Albuquerque, New Mexico 87185, USA.*

[2]*Center for Integrated Nanotechnologies, Sandia National Laboratories, Albuquerque, New Mexico 87185, USA.*

[3]*Max Planck Institute for the Science of Light, 91058 Erlangen, Germany.*

[4]*Friedrich-Alexander-Universität Erlangen-Nürnberg, 91058 Erlangen, Germany.*

*e-mail: ibrener@sandia.gov

Table of Contents

Supplementary Note 1: Multipolar decomposition analysis of the quasi-bound states in the continuum (qBICs) modes.
Supplementary Note 2: Metasurface Fabrication.
Supplementary Note 3: Dimensions of the metasurfaces.
Supplementary Note 4: Optical properties of metasurfaces at the pump wavelength
Supplementary Note 5: Experimental Setups
Supplementary Note 6: Far-Field polarization states of the ED-qBIC and in-plane Mie-type modes.
Supplementary Note 7: Two-photon state and the effect of the polarizer.
Supplementary Note 8: Fitting Lorentzian Functions to Experimental Data for Quantum Interference.
Supplementary Note 9: SPDC spectra with varied pump polarization while maintaining photon distinguishability.

Supplementary Note 1: Multipolar decomposition analysis of the quasi-bound states in the continuum modes

We carried out a thorough multipolar decomposition analysis to understand the origins of the resonances linked to electric dipole (ED) and magnetic dipole (MD) qBICs mentioned in the main text. For this analysis, we focus on QOM-B since QOM-A is primarily a variation in size of the resonator. Using Ansys Lumerical finite-difference time-domain (FDTD) simulations, we simulated the fields in the metasurface, which was excited by a plane wave with its polarization set at 135° tilted from $[\bar{1}10]$ toward [001] direction (see Fig. 1b). We also utilized the open-source package MENP (Multipole Expansion for Nanophotonics), which is based on the exact multipolar expansion method, to provide a detailed breakdown of the multipole contributions [S1]. This analysis, as shown in Fig. S1, confirms that the qBICs we identified as ED-qBIC and MD-qBIC indeed exhibit electric dipole and magnetic dipole characteristics, respectively.

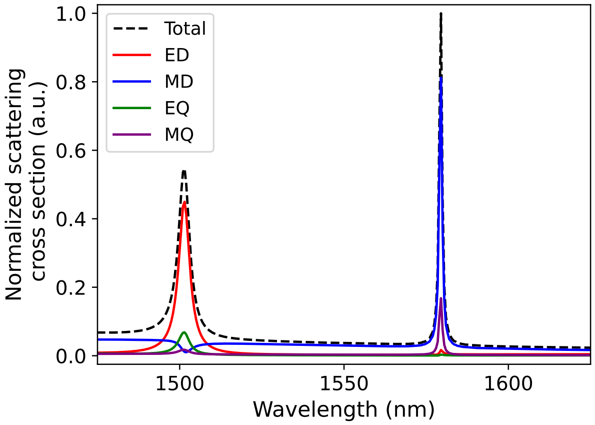

FIG. S1. **Multipolar decomposition analysis of QOM-B.** Normalized scattering cross-section spectra for QOM-B obtained through multipolar decomposition, with the metasurface in the simulation excited by a plane wave whose incident polarization is oriented at 135° tilted from $[\bar{1}10]$ toward [001] (see Fig. 1b). The analysis considered the four lowest-order multipole moments: electric dipole (ED), magnetic dipole (MD), electric quadrupole (EQ), and magnetic quadrupole (MQ).

Supplementary Note 2: Metasurface Fabrication

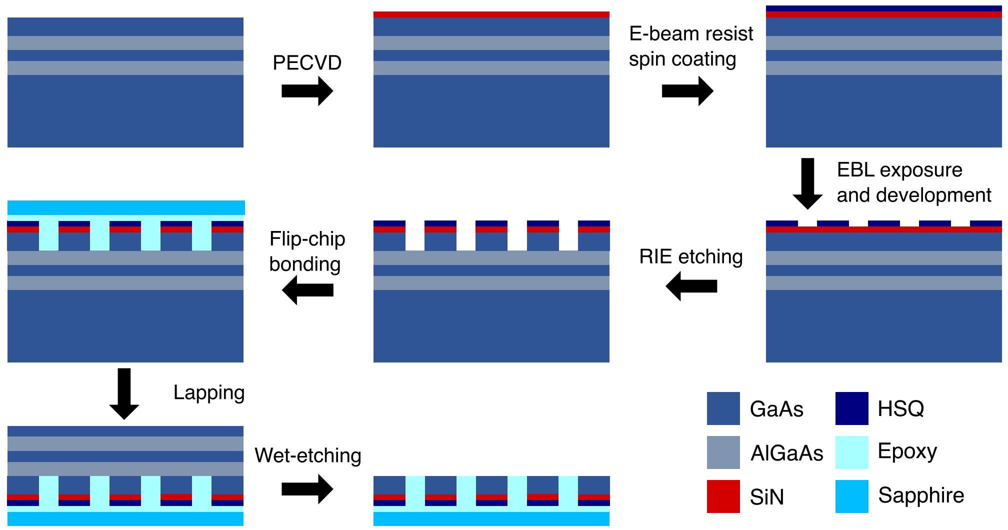

FIG. S2. **Fabrication process.** Fabrication process for [110]-oriented GaAs metasurface.

Supplementary Note 3: Dimensions of the metasurfaces

**Metasurfaces**	**Cube length, a (nm)**	**Notch width, w (nm)**	**Notch length, l (nm)**	**Period (nm)**
QOM-A	376	118	196	795
QOM-B	351	107	175	744

TABLE S1. Dimensions of the metasurfaces

Supplementary Note 4: Optical properties of metasurfaces at the pump wavelength

In designing the metasurface, we focused on positioning qBICs at the Γ point of the lowest-order bands, specifically at double the CW pump wavelength. However, the resonant modes at or near the CW pump wavelength were not considered, and the presence of multiple modes in that vicinity results in the excited mode being a mixture of these resonances.

We studied the optical properties of metasurfaces in the vicinity of the pump wavelength, focusing only on QOM-B since QOM-A is primarily a variation in size of the resonator. In Figs. S3a and b, we present experimentally measured transmission spectra from 500 nm to 1000 nm and corresponding Lumerical simulation results, respectively. The specific features do not match exactly, which we attribute to fabrication imperfections and to the fact that multiple modes contribute simultaneously, unlike around double the pump wavelength, where only a few modes are present. Furthermore, we performed the multipolar decomposition analysis, as shown in Fig. S3c. No single multipole component dominates near the pump wavelength, indicating the presence of multiple modes. These results suggest that there is potential for improvement by designing high-Q modes at both the pump and signal/idler wavelengths of the SPDC process, which would enhance the overlap integral of the fields at these wavelengths.

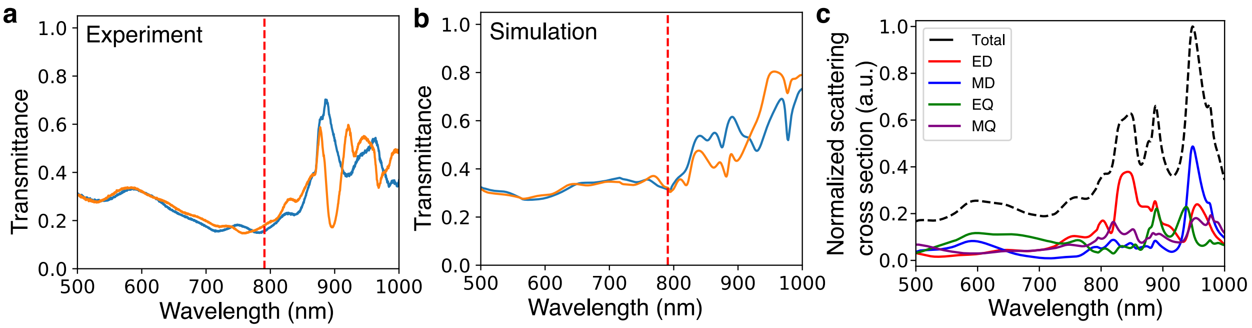

FIG. S3. **Optical properties of QOM-B at the pump wavelength.** **a** Measured white-light transmission spectra of QOM-B and **b** and corresponding simulation results using Lumerical for incident polarizations along the direction 45° (blue) and 135° (orange) tilted from [$\bar{1}$10] toward [001] (see Fig. 1b), respectively. Red dashed lines indicate the wavelength of the pump beam. **c** Normalized scattering cross-section spectra for QOM-B obtained through multipolar decomposition, where the metasurface in the simulation is excited by a plane wave with the polarization tilted by 135° from [$\bar{1}$10] toward [001].

Supplementary Note 5: Experimental Setups

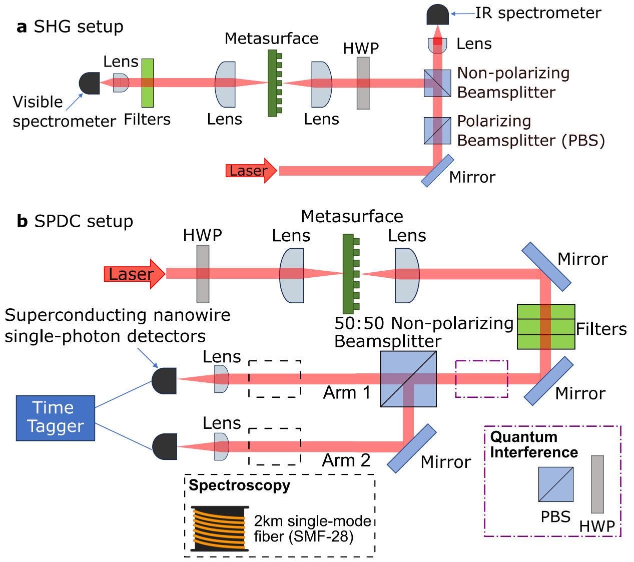

FIG. S4. **Schematics of experimental setups.** **a** Schematics of SHG setup. A pulsed laser (350 fs, 1MHz) pumps the GaAs metasurfaces from the air side. The emitted SHG radiation is filtered off the pump laser using a shortpass filter, and measured with visible spectrometer. The transmission port of the non-polarizing beam splitter (90% transmission, 10% reflection) is used to record the pump intensity in an infrared (IR) spectrometer. **b** Schematics of SPDC setup. A continuous-wave laser centered at 788.4 nm pumps the GaAs metasurfaces from the substrate side. The emitted SPDC radiation is filtered off the pump laser using a combination of longpass filters, and then sent to a Hanbury-Brown-Twiss-like interferometer for further analysis and detection. For spectroscopic measurements 2km single-mode fibers are added at the positions indicated with dashed boxes, and for quantum interference measurements additional optical components are further added at the position indicated with dash-dotted box.

Supplementary Note 6: Far-Field polarization states of the ED-qBIC and in-plane Mie-type modes in QOM-A

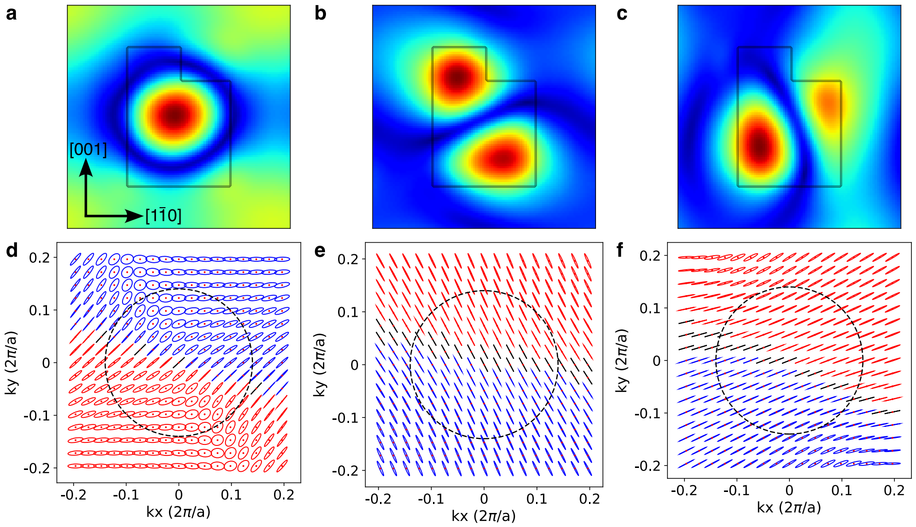

FIG. S5. **Far-field polarization states in QOM-A. a** The cross-sectional electric fields calculated using guided-mode expansion (GME) simulations at the center of the meta-atom for ED-qBIC, and **b-c** the two low-Q in-plane MD Mie modes in QOM-A, respectively. **d** The far-field polarization states of the metasurface calculated using GME simulations for the bands on which ED-qBIC and **e-f** the in-plane modes lie at the Γ point, respectively. Dashed circles indicate the range in k-space covered by the effective collection NA of ∼0.14, and a is the period of the lattice.

Supplementary Note 7: Two-photon state and the effect of the polarizer

We assume the ED-qBIC and in-plane Mie-type modes drive independently two SPDC processes when pumped coherently by a continuous-wave laser centered at frequency ω_p. The quantum state generated via SPDC driven by the ED-qBIC mode is

$$|\mathrm{qBIC}\rangle = \int d\omega A(\omega)|\omega, \vec{P}_1\rangle \otimes |\omega_p - \omega, \vec{P}_1\rangle, \tag{S1}$$

where $A(\omega)$ is a function that models the spectral range where the zero-point vacuum fluc-

tuations -the density of states- are enhanced by the ED-qBIC mode. $\vec{P}_1$ is the polarization state of the emitted photons as given by the far-field polarization state of the ED-qBIC mode (see Fig. S5d). Likewise,

$$|\text{Mie}\rangle = \int d\omega B(\omega)|\omega, \vec{P}_2\rangle \otimes |\omega_p - \omega, \vec{P}_2\rangle, \tag{S2}$$

is the quantum state generated via SPDC driven by the in-plane Mie-type mode, where B_ω gives the spectral range where the Mie-type mode enhances the zero-point vacuum fluctuations. Since the ED-qBIC mode has a larger Q factor, the function $A(\omega)$ is narrower than $B(\omega)$. Here, $\vec{P}_2$ is the far-field polarization state of the in-plane Mie-type modes (Figs. S5e-f).

The quantum state of the composite system is given then by

$$|\Psi\rangle = \alpha|\text{qBIC}\rangle + \beta|\text{Mie}\rangle, \tag{S3}$$

where α and β are complex coefficients that satisfy the normalization condition $|\alpha|^2 + |\beta|^2 = 1$. Inserting Eqs. S1 and S2 into Eq. S3 gives

$$|\Psi\rangle = \int d\omega \left[A(\omega)|\omega, \vec{P}_1\rangle \otimes |\omega_p - \omega, \vec{P}_1\rangle + B(\omega)|\omega, \vec{P}_2\rangle \otimes |\omega_p - \omega, \vec{P}_2\rangle\right], \tag{S4}$$

where we have absorbed the coefficients α and β into the functions $A(\omega)$ and $B(\omega)$, respectively. These functions, in addition to including the spectral dependence, also contain important parameters proportional to the conversion efficiency, such as the effective second-order susceptibility. Eq. S4 is the quantum state of the system just before the polarizer.

Now, let $\hat{P}_\theta \otimes \hat{P}_\theta$ be the operator of the polarizer acting on the Hilbert spaces of the signal and idler photons, where θ is the orientation of the polarizer. In our experiment, θ is twice the angle between the fast axis of the HWP and the transmission axis of the PBS (see Fig. S4b). The quantum state of the system after the polarizer is given by

$$\begin{aligned} |\Psi'\rangle &= \hat{P}_\theta \otimes \hat{P}_\theta|\Psi\rangle \\ &= \int d\omega \left[A(\omega)f(\theta) + B(\omega)g(\theta)\right] |\omega, \vec{P}_\theta\rangle \otimes |\omega_p - \omega, \vec{P}_\theta\rangle, \end{aligned} \tag{S5}$$

where $\vec{P}_\theta$ is the polarization direction on which the operator $\hat{P}_\theta \otimes \hat{P}_\theta$ is projecting the state,

and the functions $f(\theta)$ and $g(\theta)$ give the projections of $|\vec{P}_1\rangle$ and $|\vec{P}_2\rangle$ onto $|\vec{P}_\theta\rangle$, respectively, for the biphotons, that is,

$$f(\theta) = \langle \vec{P}_\theta | \vec{P}_1 \rangle \langle \vec{P}_\theta | \vec{P}_1 \rangle, \tag{S6}$$

and similarly for $g(\theta)$. In the experiment, we can balance the contributions of the ED-qBIC and in-plane Mie-type modes after the PBS by adjusting the angle θ.

Supplementary Note 8: Fitting Lorentzian Functions to Experimental Data for Quantum Interference

To capture the fundamental indistinguishability of biphotons according to Feynman's indistinguishability criterion, we employed the modulus square of the combined amplitudes of two Lorentzian functions, which can be expressed mathematically as follows:

$$I(\lambda) = \left| \frac{A_1 \left(\frac{\Gamma_1}{2}\right)^2}{\pi \left((\lambda - \lambda_1)^2 - \left(\frac{\Gamma_1}{2}\right)^2 \right)} - e^{i\phi} \frac{A_2 \left(\frac{\Gamma_2}{2}\right)^2}{\pi \left((\lambda - \lambda_2)^2 - \left(\frac{\Gamma_2}{2}\right)^2 \right)} \right|^2, \tag{S7}$$

where fitting parameters $A_{1,2}$, $\lambda_{1,2}$ and $\Gamma_{1,2}$ are amplitudes, center wavelengths and full-widths at half-maximum of two Lorentzian functions corresponding to the qBIC and the in-plane Mie mode, respectively, and ϕ is the phase between them. ϕ from the fit, which is shown as the blue dashed curve in Fig. 4c, is precisely π and other fitting parameters are shown in Table S2.

Modes	Center Wavelength (nm)	Full-width half-maximum, FWHM (nm)	Amplitude (a.u.)
qBIC	1581.1 ± 0.2	14.2 ± 0.9	33 ± 2
In-plane Mie mode	1450 ± 40	400 ± 60	380 ± 80

TABLE S2. The fitting parameters for the blue dashed curve in Fig. 4**c**.

Note that the two functions on the right-hand side of Eq. S7 are precisely the terms inside the square brackets in S5.

Furthermore, we conducted the fitting for the non-optimal cases where the filtered polarization angles are at 0° and 45° as shown in Figs. 4a and b. However, it is anticipated that the low-Q component in the fit to the interference of two Lorentzian-shaped functions will exhibit a larger error and to address this, we fixed the fit parameter for the center wavelength of the low-Q component to the corresponding value obtained in the fit to the optimal case (i.e. Fig. 4c), where the fit provided an excellent representation of the measured data. The resulting fitting parameters are shown in Table S3, and they indicate that FWHM of the two modes are relatively consistent. These results reinforce that the variations in amplitude and phase are crucial in determining the specific lineshape observed in each example.

Filtered polarization angles	Modes	FWHM (nm)	Amplitude (a.u.)	Relative phase (π rad.)
0°	qBIC	9 ± 4	10 ± 9	1.7
	In-plane Mie mode	500 ± 40	490 ± 30	
45°	qBIC	9.4 ± 0.6	15 ± 2	0.5
	In-plane Mie mode	500 ± 50	260 ± 20	

TABLE S3. The fitting parameters for the blue dashed curve in Figs. 4**a** and **b**, obtained by fitting Eq. S7, with the center wavelength of the low-Q component fixed at the value determined from Fig. 4**c**.

Supplementary Note 9: SPDC spectra with varied pump polarization while maintaining photon distinguishability

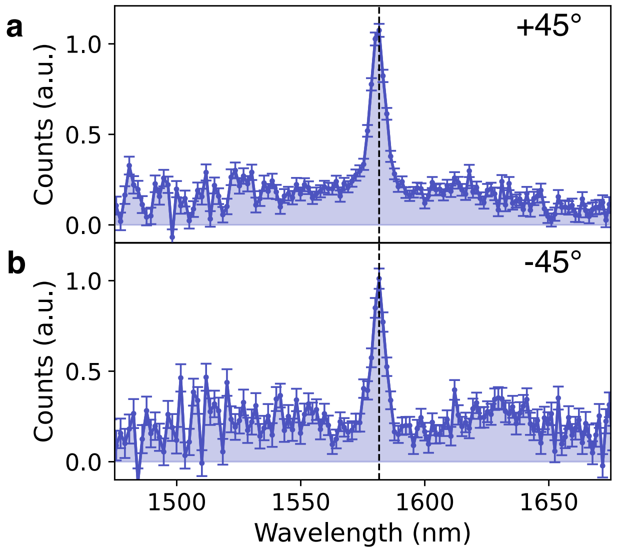

FIG. S6. **SPDC spectra of distinguishable biphotons.** **a** Measured SPDC spectra from QOM-A when the linear polarization of the pump beam was rotated 45° and **b** -45° with respect to that in Fig. 3**c**, where the pump polarization was selected to optimize the nonlinear interaction. Changing the polarization of the pump beam also changes the ratio between the contributions from qBIC and in-plane Mie resonances but still preserves the distinguishability of the photon polarizations. Therefore, unlike in Fig. 4**c**, the Fano contour, with a clear dip approaching zero, cannot be observed in the SPDC spectra. Black dashed lines indicate double the wavelength of the pump beam.

References

[S1] T. Hinamoto and M. Fujii, MENP: an open-source MATLAB implementation of multipole expansion for nanophotonics, OSA Continuum **4**, 1640 (2021).
